# Editorial: Update on Vascular Contributions to Age-Related Neurodegenerative Diseases and Cognitive Impairment - Research of ISNVD 2020 Meeting

**DOI:** 10.3389/fneur.2021.797486

**Published:** 2021-11-11

**Authors:** Yulin Ge, Robert Zivadinov, Meiyun Wang, Andreas Charidimou, E. Mark Haacke

**Affiliations:** ^1^Department of Radiology, New York University (NYU) Grossman School of Medicine, New York, NY, United States; ^2^Department of Neurology, Jacobs School of Medicine and Biomedical Sciences, Buffalo, NY, United States; ^3^Department of Radiology, Henan Provincial People's Hospital, Zhengzhou, China; ^4^Department of Neurology, Boston University Medical Center, Boston University School of Medicine, Boston, MA, United States; ^5^Department of Radiology, Wayne State University School of Medicine, Detroit, MI, United States

**Keywords:** MRI, cerebral blood flow, stroke, dementia, neurodegeneration, multiple sclerosis, Parkinson's disease

Vascular pathologies are among the most common contributors to neurodegenerative changes along the spectrum of normal aging to dementia. Cerebral small vessel disease (SVD) in particular represents a diverse range of neuropathological conditions that affect capillaries, small arteries, arterioles and small veins in the brain and have a prominent role as vascular contributions to cognitive impairment and dementia (VCID) (1, 2), even in patients considered to have Alzheimer's disease. These cerebrovascular abnormalities are associated with imaging based biomarkers, including white matter hyperintensities, lacunes, cerebral microbleeds, enlarged perivascular spaces, cerebral microinfarcts, and cortical atrophy. As such, SVD currently causes 20% of ischemic strokes, are the underlying cause of spontaneous intracerebral hemorrhage and constitutes a main source of cognitive decline or VCID, particularly in the elderly (3). It has been reported that with one in three people having clinical ischemia or stroke during their lifetime, dementia occurs in an estimated 30% of post-stroke patients; the effects on public health impact is enormous. However, apart from risk containment, efforts to prevent or to treat SVD are ineffective; much remains to be learned about the underpinnings of vascular pathophysiology, regional vulnerability, and disease progression over time. Along this line, MRI plays an important role due to its potential of revealing *in vivo* pathophysiology, early detection and clinical correlations.

In this Research Topic, we aim to gather recent research to better understand vascular contributions to age-related neurodegenerative diseases and cognitive impairment. This special issue includes 2 reviews, 2 brief research reports, and 11 original research articles. The articles cover a number of neurodegenerative diseases including stroke, Alzheimer's disease (AD), Parkinson's disease (PD), and multiple sclerosis (MS) and give insight into ongoing topics on both arterial (e.g., ischemia) and venous (e.g., inflammation) systems as well as the glymphatic system. We also aim to promote research exchange across different fields (e.g., imaging, vascular anatomy, brain physiology, clinical symptom monitoring and treatment). Most papers in this special issue applied MRI studies to better understand disease mechanisms, to help differential diagnosis, and to develop imaging biomarkers for evaluating disease progression. New technical developments of advanced neurovascular imaging in age-related cognitive impairment are also included.

Currently, there is still a significant knowledge gap of how the vascular pathophysiological underpinnings initiate the long-term neurodegeneration processes in age-related cognitive impairment. There are seven papers on this topic from researchers with diverse expertise. Post-stroke cognitive impairment (PSCI) occurs in over 80% of acute stroke survivors and represents one of the major causes of vascular dementia, however, its underlying mechanism is unknown. Zhong et al. reported that in patients with early subacute ischemic stroke, the lesion volume in the cortical cholinergic pathways (CCP) was associated with cognitive impairment. The disruption of the cholinergic pathways might contribute to newly developed dementia in patients with PSCI. Sharma et al. (4) further demonstrated that the PSCI changes can occur 4-8 weeks post-infarct, and patients with high-risk of early PSCI development should be identified for targeted rehabilitation and counseling to improve longer-term outcomes. Savadori et al. (5) assessed a cohort of elderly who had atrial fibrillation (AF), a condition associated with reduced physical performance and increased risk of cognitive decline, suggesting a possible link between motor and cognitive performance. Cerebral SVD is commonly seen in the elderly. One study by Zhao et al. investigated whether spatial navigation performance is impaired in elderly SVD patients. Compared to healthy controls, only severe SVD or patients with higher Fazekas scores exhibited significantly worse performance on simulated navigation tasks, suggesting spatial navigation decline may be one of manifestation in severe SVD. To study the genetic role in subcortical vascular cognitive impairment (svMCI), Yoon et al. compared the longitudinal changes in SVD markers and cognitive function between svMCI patients with and without NOTCH3 variant, and they showed NOTCH3(+) svMCI group had much greater increases in the lacune and cerebral microbleed counts than the NOTCH3(–) svMCI group. Over 5 years follow up, however, no significant differences were found between the two groups regarding dementia conversion rate in these patients.

Regarding screening for cognitive impairment after stroke, Xu et al. (6) compared the diagnostic accuracy of a Chinese version of the Quick Mild Cognitive Impairment (Qmci-CN) with a widely-used Chinese version of Montreal Cognitive Assessment (MoCA-CN) and Mini-Mental State Examination (MMSE-CN). They showed that the Qmci-CN is accurate in identifying post-stroke dementia and separating PSD from no-dementia PSCI. This test was comparable to MoCA-CN but with a significantly shorter administration time. However, the Qmci-CN is relatively poor in differentiating no-dementia PSCI patients from controls. One animal study by Wei et al. investigated the age effects on cerebrovascular and physiological parameters by characterizing the longitudinal time courses across the adult lifespan in mice. The results revealed an age-related increase in oxygen consumption without decrease of cerebral perfusion (unlike human studies), which is consistent with the absence of white matter hyperintensities (WMHs) in aged mice. A more relevant implication from this study is that using animal models to study human aging process has limitations.

Recently there is substantial interest regarding the role of the glymphatic system in AD. Agarwal and Carare (7) provided a novel overview of the role of cerebral vessels and their connection with cerebrospinal fluid (CSF) and interstitial fluid (ISF) in brain waste clearance. Emerging evidence shows damage to macro/microvasculature or their dysfunction will compromise the fluid movement and alter the homeostasis of the brain, which in turn leads to neuronal cell loss and dementia. Since the central nervous system (CNS) is completely submerged in CSF, it is critical to map out its clearance pathway from peri-neural and peri-vascular space to bulk flow in subarachnoid space. Using several fluid-sensitive MRI techniques, Fahmy et al. (8) successfully demonstrated the presence of CSF within all peri-neural (cranial and spinal nerves) and peri-vascular spaces *in vivo* human brain and spinal cord. These findings suggest that anatomically, substance exchange neural tissue and outside glymphatic space can only occur through CSF and vascular pathways although further investigations are warranted to study their specific role in waste clearance and immunity.

Two papers focused on brain perfusion changes in Parkinson's disease. Laganà et al. applied a multimodal MRI approach to investigate early PD with resting state functional MRI (rsfMRI) and arterial spin labeling (ASL). They found reduced functional connectivity (FC) in patients within a sensory-motor network and visual networks accompanied by a decreased CBF compared to controls. Another study by Pelizzari et al. investigated CBF within the regions associated with fronto-parietal network in PD patients without dementia. They found significantly lower CBF in the left superior and inferior parietal lobes in patients who also performed poorer on MoCA tests. The decreased perfusion in parietal regions may be associated with lower visuomotor skills and has potential for longitudinal studies investigating cognitive decline in PD.

Haacke et al. reviewed over 200 papers and provided an in-depth discussion of the evidence for vascular pathogenesis in MS lesions. They put together multiple key pieces of information from the literature regarding vascular remodeling, venous collagenosis, abnormal venous flow, perfusion, endothelial dysfunction and vascular endothelial growth factors. After combining their own findings using ultra-small superparamagnetic iron oxide or USPIO-enhanced MRI, they raised one possible theory on MS lesion etiology that is associated with locally disrupted blood flow, which in turn leads to remodeling of the medullary veins followed by endothelial damage with the subsequent cascade of inflammatory and demyelinating events. Two other papers reported cerebrovascular abnormalities in MS. Jakimovski et al. (9) investigated the associations between cerebral perfusion and disease outcomes in MS patients with and without comorbid cardiovascular diseases (CVD). They found decreased brain perfusion in both cortical and deep GM is associated with poorer MS outcomes, but not with the presence of CVD. By combining plasma and MRI biomarkers of MS, Ziliotto et al. (10) investigated the link between microvascular abnormalities and immune inflammatory changes and their role in neurodegeneration. They showed that higher protein C (PC) levels were associated with large brain volume loss in relapsing remitting but not in progressive MS. Higher chemokine C-C motif ligand 18 (CCL18) levels were associated with higher T2-lesion volumes in all MS patients, and higher CCL18 levels were associated with lower volumes of the GM in progressive MS. These results will help us to better understand the disease heterogenetic nature of MS.

Regarding the novel cerebrovascular imaging techniques for aging and dementia studies, Taneja et al. (11) compared two quantitative CBF techniques, phase-contrast (PC)- and ASL-based hypercapnia MRI that were used to assess cerebrovascular reactivity (CVR). CVR is a relatively new marker for assessing vasomotor function and has shown great promise in predicting age-related neurodegeneration. The results suggest that PC-based CVR is a more sensitive method for aging effects despite being a global measure and lacking spatial information. This voxel-wise ASL-based method tends to underestimate CVR.

In summary, this is an exciting time in neurovascular and aging research. In this Research Topic, we hope to provide a comprehensive collection to cover the latest advances with a wide range of cross-disciplinary topics on neurovascular research in neurodegenerative diseases.

## Author Contributions

YG took the lead in writing this editorial. All authors provided critical feedback and helped shape the manuscript.

## Funding

This work is supported by National Institute of Health grants (RF1 NS11041, R01 NS108491, and R13 AG067684). This study is also supported by Alzheimer's Association (AARG-17-533484).

## Conflict of Interest

The authors declare that the research was conducted in the absence of any commercial or financial relationships that could be construed as a potential conflict of interest.

## Publisher's Note

All claims expressed in this article are solely those of the authors and do not necessarily represent those of their affiliated organizations, or those of the publisher, the editors and the reviewers. Any product that may be evaluated in this article, or claim that may be made by its manufacturer, is not guaranteed or endorsed by the publisher.
